# p53 regulates lysosomal membrane permeabilization as well as cytoprotective autophagy in response to DNA-damaging drugs

**DOI:** 10.1038/s41420-022-01293-x

**Published:** 2022-12-29

**Authors:** Gai Yamashita, Naoharu Takano, Hiromi Kazama, Kiyoaki Tsukahara, Keisuke Miyazawa

**Affiliations:** 1 Department of Otorhinolaryngology, Head and Neck Surgery, Tokyo Medical University Hospital, Shinjuku-ku, Tokyo, 160-0023 Japan; 2 Department of Biochemistry, Tokyo Medical University, Shinjuku-ku, Tokyo, 160-8402 Japan

**Keywords:** Cell death, Macroautophagy, Chemotherapy, Cancer therapeutic resistance

## Abstract

Lysosomes are single-membraned organelles that mediate the intracellular degradation of macromolecules. Various stress can induce lysosomal membrane permeabilization (LMP), translocating intralysosomal components, such as cathepsins, to the cytoplasm, which induces lysosomal-dependent cell death (LDCD). This study reports that p53 regulates LMP in response to DNA-damaging drugs. Treating wild-type *TP53* A549 cells with DNA-damaging drugs (namely, doxorubicin, carboplatin, and etoposide) induced LMP and accelerated cell death more rapidly than treating *TP53*-knockout (KO) A549 cells. This suggested p53-dependent LMP and LDCD induction in response to DNA damage. LMP was induced by p53-dependent BID upregulation and activation, followed by translocation of truncated BID to lysosomes. Simultaneously, autophagy for damaged lysosome elimination (lysophagy) was activated via the p53–mTOR–TEFB/TFE3 pathways in response to DNA damage. These data suggested the dichotomous nature of p53 for LMP regulation; LMP induction and repression via the p53–BID axis and p53–mTOR–TFEB/TFE3 pathway, respectively. Blocking autophagy with hydroxychloroquine or azithromycin as well as *ATG5* KO enhanced LMP and LDCD induction after exposure to DNA-damaging drugs. Furthermore, lysosomal membrane stabilization using U18666A, a cholesterol transporter Niemann-Pick disease C1 (NPC1) inhibitor, suppressed LMP as well as LDCD in wild-type *TP53*, but not in *TP53*-KO, A549 cells. Thus, LMP is finely regulated by TP53 after exposure to DNA-damaging drugs.

## Introduction

Lysosomes contain more than 60 hydrolytic enzymes and are maintained between pH 4.5 and 5. They degrade macromolecules transported by endocytosis, phagocytosis, and autophagy [1, 2]. Recently, lysosomes have attracted attention as organelles regulating various functions such as signal transduction, cancer metastasis, immune response, plasma membrane repair, and vesicle exocytosis [3]. Lysosomes are widely localized throughout the cytoplasm and can be categorized into two major groups: an immobile population near the microtubule organizing center in the vicinity of the nucleus and a highly mobile population in the periphery near the plasma membrane [3]. Lysosomes around the nucleus mainly fuse with autophagosomes to form autolysosomes, which are involved in the degradation of their contents. However, lysosomes located in the periphery regulate plasma membrane repair and activate the mammalian target of rapamycin complex I (mTORC1) [4–6]. Activated mTORC1 on the lysosomal surface promotes global protein synthesis by phosphorylating S6 kinase and 4EBP1, which regulate translation initiation and induce cell growth [7]. Thus, lysosomes regulate the catabolic pathway through autophagy as well as the anabolic pathway through mTORC1 activation. Autophagy is involved in tumor growth promotion and chemotherapeutic drug resistance [8]. Therefore, current studies on cancer therapy have focused on autophagy inhibition. Previous studies have reported that the co-administration of macrolide antibiotics, particularly autophagy-inhibiting azithromycin (AZM), and tyrosine kinase inhibitors (TKIs) or proteasome inhibitors induce cytoprotective autophagy, enhancing cell death induction in various cancer cell lines, including multiple myeloma cells and pancreatic cancer cells [9–11].

Lysosomal membrane permeabilization (LMP) is the process by which various stress types damage the lysosome, thus causing lysosomal swelling and leakage of lysosomal contents into the cytoplasm [1, 12, 13]. Lysosomotropic compounds, reactive oxygen species, and BAX, a pro-apoptotic Bcl-2 member protein cause LMP [14]. LMP causes hydrolytic enzyme release into the cytoplasm, cytoplasmic acidification, lysosomal function loss, and in more severe cases, BID- and BAX-induced apoptosis or other types of cell death [15]. Such LMP-induced cell death is called lysosome-dependent cell death (LDCD).

In response to lysosomal stress, endosomal sorting complexes required for transport (ESCRT) repair the membrane in response to lysosomal membrane damage [16]. When the lysosomal membrane is too damaged to repair, lysophagy is induced to degrade and eliminate the damaged lysosomes [17]. Simultaneously, the depleted lysosomes are replaced by newly synthesized lysosomes by the mTORC1–TFEB pathway: the transcription factor TFEB is the master regulator for lysosomal biogenesis. TFEB is usually phosphorylated by mTORC1 localized on the lysosome membrane, and anchored to 14-3-3 protein in the cytoplasm. In response to LMP induction, mTORC1 dissociates from the lysosomal membrane, thus dephosphorylating and translocating the TFEB nucleus. This induces lysosomal biogenesis and autophagosome formation for cytoplasmic lysosome homeostasis [18].

Previous studies have demonstrated that DNA-damaging anticancer drugs induce LMP and also enhance cell death by damaged lysosome accumulation after co-administration with AZM, which inhibits autophagy. Interestingly, the cell death was not enhanced in *TP53*-mutated or -knock out (KO) cells. This suggests that LMP induction is p53-dependent [19]. However, the mechanism of DNA-damaging drug-mediated autophagy induction and p53-dependent LMP induction remains unclear. This study investigates the underlying molecular mechanism of p53-dependent LMP and autophagy induction.

## Results

### DNA-damaging drugs induce LMP and accelerate cell death p53-dependently

**Fig. 1:**
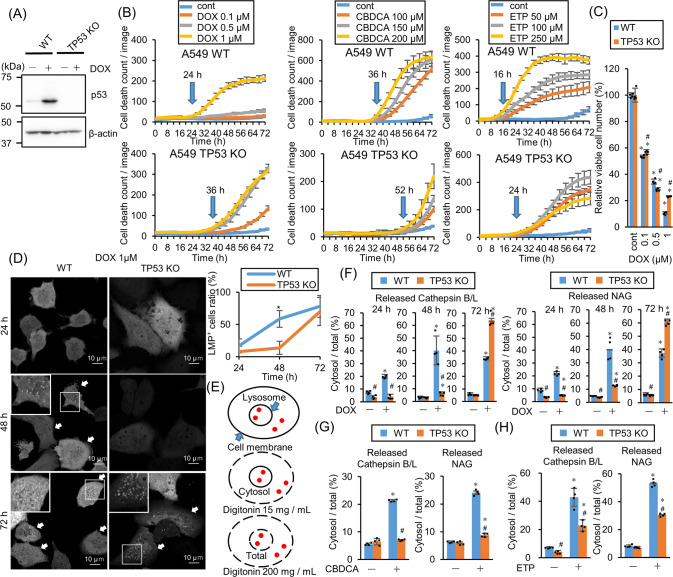
*TP53*-knockout (KO) retards lysosomal membrane permeabilization (LMP)-mediated cell death in response to DNA-damaging drugs. **A** *TP53*-KO in A549 cells was confirmed by western blotting. p53 expression was assessed after 1 µM doxorubicin (DOX) treatment for 24 h. β-actin was used as a control. **B** Wild-type (WT) and *TP53*-KO A549 cells were treated with DOX, etoposide (ETP), or carboplatin (CBDCA) and dead cell count was monitored every 4 h by a live-cell imaging system with propidium iodide (PI) staining. The cell death initiation point after 1 µM DOX treatment is indicated using blue arrows. The cell death initiation point was defined as follows: the highest and lowest dead cell count under drug treatment was defined as 100 and 0, respectively. Cell death onset was defined as the point where the difference between the control and drug-treated dead cell counts was >6. **C** The viable WT and *TP53*-KO A549 cell count 48 h after DOX treatment. *n* = 4, bar = mean ± SD, *p < 0.05 vs. cont., #*p* < 0.05 vs. WT. **D** Aequorea coerulescens green fluorescence protein-conjugated galectin-3 (AcGFP–Gal3)-expressing WT or *TP53*-KO A549 cells were treated with 1 µM DOX for 24, 48, or 72 h. AcGFP–Gal3 puncta were observed using confocal microscopy and the LMP^+^ cell ratio was calculated and summarized in right. Arrows indicate LMP^+^ cells. Scale bar = 10 µm. *n* = 10, bar = mean ± SD, *p < 0.05 vs. *TP53*-KO. **E** A schema of LMP enzymatic assay. The plasma membrane and lysosomal membrane are shown in black. Lysosomal enzymes are shown in red dots. Membranes permeabilized using different digitonin concentrations are shown with a dashed line. The LMP degree was measured by assessing the leaked cytosolic (15 mg/mL digitonin) and whole-cell (200 mg/mL digitonin) lysosomal enzymatic activity. **F** LMP in WT and *TP53*-KO A549 cells treated with 1 µM DOX for 24, 48, and 72 h were measured by assessing released cytosolic N-acetyl-glucosaminidase (NAG) or cathepsin B/L activity. *n* = 4, bar = mean ± SD, *p < 0.05 vs. cont., #*p* < 0.05 vs. WT. **G**,**H** LMP in WT and *TP53*-KO A549 cells treated with (**G**) 150 µM CBDCA for 66 h or (**H**) 250 µM ETP for 29 h were measured. *n* = 4, bar = mean ± SD, *p < 0.05 vs. cont., #*p* < 0.05 vs. WT.

To address the mechanism of p53-dependent LMP induction, *TP53*-KO A549 cells were established using the CRISPR–Cas9 system (Fig. 1A). Cell death induction was monitored by propidium iodide (PI) staining with the live-cell imaging system after treatment with the DNA-damaging anticancer drugs doxorubicin (DOX), carboplatin (CBDCA), and etoposide (ETP). As we reported previously, treatment with all drugs delayed *TP53*-KO cell death (Fig. 1B) [19]. Moreover, the viable *TP53*-KO cell count after 1 μM DOX treatment for 48 h was higher than the viable wild-type (WT) cell count (Fig. 1C). To assess the involvement of LMP in cell death induction kinetics, galectin-3 (Gal3) puncta assay was performed using A549 cells stably expressing Aequorea coerulescens green fluorescence protein-conjugated Gal3 (AcGFP–Gal3), where AcGFP–Gal3 accumulated in the damaged lysosomes and showed a patchy pattern [20]. AcGFP–Gal3 colocalized with lysosomal protein LAMP2 after LMP induction using L-leucyl-L-leucine methyl ester (LLOMe) treatment (Fig. S1). Interestingly, *TP53*-KO and WT cells were Gal3-positive 72 and 48 h after DOX treatment, respectively (Fig. 1D). LMP was also evaluated by measuring the cytoplasmic leakage of lysosomal enzymes, namely cathepsins and N-acetyl-glucosaminidase (NAG). The cells were treated with different concentrations of digitonin, a detergent that permeabilizes only plasma membrane at a low concentration and both plasma membrane and lysosomal membrane at a high concentration, to evaluate lysosomal enzyme leakage (Fig. 1E) This assay system was more sensitive than Gal3 puncta assay and detected LMP 24 h after DOX treatment (Fig. 1F). CDBCA and ETP treatment also induced LMP in WT cells but the induction was significantly suppressed in *TP53*-KO cells (Fig. 1G, H). These data show that cell death induction and LMP were delayed in *TP53*-KO cells as compared with WT cells. Thus, LMP was induced p53-dependently.

### DNA-damaging drugs induce autophagy via the p53–mTOR–TFEB pathway, repressing LMP

**Fig. 2:**
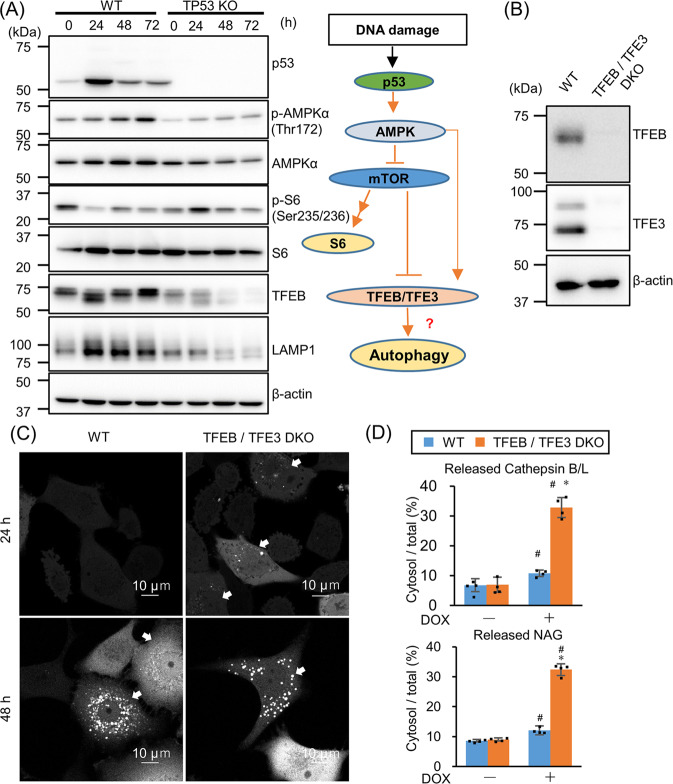
The p53–mTOR pathway activates TFEB and suppresses lysosomal membrane permeabilization (LMP). **A** p53, p-AMPKα, AMPKα, p-S6, S6, TFEB, and LAMP1 levels were assessed by western blotting after treating wild-type (WT) and *TP53*-knockout (KO) A549 cells with 1 µM doxorubicin (DOX) for 24, 48, or 72 h. β-actin was used as the loading control. The schema on the right shows DNA damage-mediated TFEB/TFE3 activation via the p53–mTOR pathway. **B** *TFEB/TFE3* double knockout (DKO) in A549 cells were confirmed by western blotting. β-actin was used as the loading control. **C** Aequorea coerulescens green fluorescence protein-conjugated galectin-3 (AcGFP–Gal3)-expressing WT or *TFEB/TFE3* DKO A549 cells were treated with 1 µM DOX for 24 or 48 h. AcGFP–Gal3 puncta were observed using confocal microscopy. LMP^+^ cells are shown with arrows. Scale bar = 10 µm. **D** LMP in WT and *TFEB/TFE3* DKO A549 cells treated with 1 µM DOX for 24 h were measured by LMP enzymatic assay. *n* = 4, bar = mean ± SD, *p < 0.05 vs. cont., #*p* < 0.05 vs. WT.

Next, we tried to address LMP induction by p53. TFEB and TFE3, the master transcriptional factors regulating lysosomal gene expression, are reported to induce LMP in ETP-treated murine fibroblast cells [21]. Since TFEB and TFE3 are activated via the p53–mTOR pathway, the expression of mTOR upstream and downstream proteins were assessed (Fig. 2A). DOX treatment increased AMPKα phosphorylation (active form of AMPKα) and suppressed S6 kinase phosphorylation in WT A549 cells, indicating mTOR inhibition in response to DOX treatment. TFEB phosphorylation by mTOR suppresses nuclear translocation of TFEB, whereas phosphorylation of TFEB by AMPK activates its transcriptional activity [22]. TFEB size decreased, accompanied by S6 kinase dephosphorylation in DOX-treated WT cells within 24 h, and then increased accompanied by AMPK phosphorylation after 48 and 72 h (Fig. 2A). This indicated TFEB activation in response to DOX treatment. Supporting this, LAMP1, one of the lysosomal proteins transcriptionally regulated by TFEB, was upregulated after DOX treatment. However, this mTOR suppression followed by TFEB activation was observed only in WT, and not *TP53*-KO cells.

To assess the involvement of TFEB/TFE3 in DOX-induced LMP, *TFEB/TFE3* double knockout (DKO) A549 cells were established using CRISPR–Cas9 system (Fig. 2B). LMP was observed earlier in TFEB/TFE3 DKO cells than in WT cells by Gal3-puncta assay (Fig. 2C). Lysosomal enzyme leakage assay also indicated that *TFEB/TFE3* DKO cells were more sensitive for LMP induction than WT cells after 24 h DOX treatment (Fig. 2D). These data suggest that TFEB/TFE3 suppresses LMP; therefore *TEFB/TFE3* DKO accelerates LMP.

**Fig. 3:**
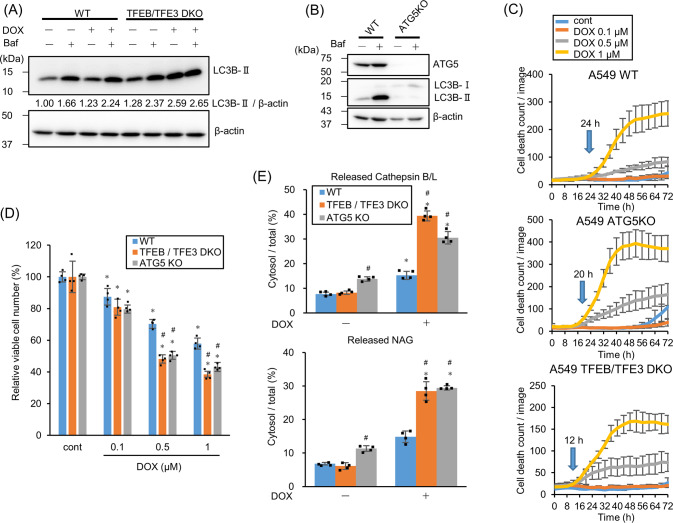
TFEB/TFE3 induces autophagy and suppresses lysosomal membrane permeabilization (LMP) to protect cells from doxorubicin (DOX)-induced cell death. **A** LC3B expression in wild-type (WT) or *TFEB/TFE3* double knockout (DKO) A549 cells treated with 1 µM DOX for 24 h was assessed by western blotting in the presence or absence of 50 nM Bafilomycin A_1_ (Baf) for 2 h. β-actin was used as a control. The relative LC3B-II band intensity is shown. **B** *ATG5*-knockout (KO) was confirmed by western blotting. WT and *ATG5*-KO A549 cells were treated with/without 50 nM Baf for 24 h. β-actin was used as the loading control. **C** WT, *ATG5*-KO, and *TFEB/TFE3* DKO A549 cells were treated with DOX and the dead cell count was monitored every 4 h by live-cell imaging system with propidium iodide (PI) staining. The cell death initiation point after 1 µM DOX treatment is indicated by blue arrows. **D** The viable WT, *ATG5*-KO, and *TFEB/TFE3* DKO A549 cell count 24 h after DOX treatment. *n* = 4, bar = mean ± SD, *p < 0.05 vs. cont., #*p* < 0.05 vs. WT. **E** LMP in WT, *ATG5*-KO, and *TFEB/TFE3* DKO A549 cells treated with 1 µM DOX for 24 h were measured by assessing released cytosolic N-acetyl glucosaminidase (NAG) or cathepsin B/L activity. *n* = 4, bar = mean ± SD, *p < 0.05 vs. cont., #*p* < 0.05 vs. WT.

In addition to lysosomal gene expressions, TFEB and TFE3 also activate autophagy-related gene expressions [23]. Therefore, TFEB/TFE3 may suppress LMP via autophagy activation, leading to the removal of damaged lysosomes (lysophagy). To address this, TFEB/TFE3-dependent autophagy induction in response to DOX treatment was evaluated. Autophagy flux assay by co-administrating DOX and Bafilomycin A_1_ (Baf) shows an increased LC3B-II than that by treatment with Baf alone, which indicates autophagy induction in response to DOX (Fig. 3A). However, *TFEB/TFE3* DKO cells did not show such increment, indicating no autophagy induction. Thus, TFEB/TFE3 activates autophagy in DOX-treated cells. Next, to address whether autophagy suppresses LMP and functions cytoprotectively, the cell death and LMP were assessed using *TFEB/TFE3* DKO as well as *ATG5*-KO cells lacking autophagosome forming ability (Fig. 3B). Both cell lines showed cell death induction earlier in response to DOX treatment than WT cells did (Fig. 3C, D). Interestingly, LMP induction increased in both cells after DOX treatment for 24 h (Fig. 3E). These data suggest that autophagy induction via the p53–mTOR–TFEB/TFE3 pathway suppresses LMP.

### BAK and BAX are partially involved in the DOX-induced LMP

**Fig. 4:**
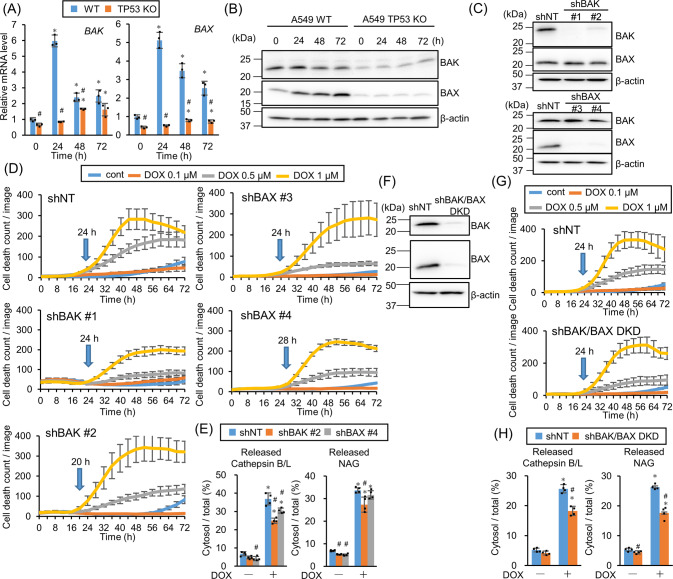
BAK and BAX are partly involved in doxorubicin (DOX)-induced lysosomal membrane permeabilization (LMP). **A** The relative *BAK* and *BAX* mRNA expression level in wild-type (WT) or *TP53*-knockout (KO) A549 cells treated with 1 µM DOX for 24, 48, or 72 h was assessed by real-time PCR. *n* = 3, bar = mean ± SD, *p < 0.05 vs. 0 h, #*p* < 0.05 vs. WT. **B** BAK and BAX expression in WT or *TP53*-KO A549 cells treated with 1 µM DOX for 24, 48, or 72 h were assessed by western blotting. β-actin was used as a loading control. **C** *BAX* and *BAK* knockdown (KD) efficiency in shRNA introduced A549 cells expressing shBAK or shBAX were confirmed by western blotting. β-actin was used as a control. **D** shNT-, shBAK #1-, #2-, or shBAX #3-, #4-expressing A549 cells were treated with DOX and the dead cell count was monitored every 4 h by live-cell imaging system with propidium iodide (PI) staining. **E** LMP in *BAK*- or *BAX*-KD A549 cells treated with 1 µM DOX for 24 h were measured by assessing released cytosolic N-acetyl glucosaminidase (NAG) or cathepsin B/L activity. *n* = 4, bar = mean ± SD, *p < 0.05 vs. cont., #*p* < 0.05 vs. shNT. **F** *BAK* and *BAX* KD efficiency in *BAK/BAX* double knockdown (DKD) cells was confirmed by western blotting. β-actin was used as a control. **G** Control or *BAK/BAX* DKD A549 cells were treated with DOX and dead cell count was monitored every 4 h by live-cell imaging system with PI staining. **H** LMP in shNT-expressing or *BAK/BAX* DKD A549 cells treated with 1 µM DOX for 24 h were measured by assessing released cytosolic NAG or cathepsin B/L activity. *n* = 4, bar = mean ± SD, *p < 0.05 vs. cont., #*p* < 0.05 vs. shNT.

This study revealed that TFEB and TFE3 suppresses LMP rather than inducing LMP. BAX and BAK belong to the Bcl-2 family, are transcriptionally regulated by p53, and form a pore on a mitochondrial outer membrane to release cytochrome c during apoptosis signaling [24, 25]. In addition, they work on the lysosomal membrane for LMP induction [26]. Real-time PCR revealed that DOX treatment upregulated *BAK* and *BAX* gene expression in WT, but not *TP53*-KO, A549 cells (Fig. 4A). Western blotting also exhibited that BAX induction attenuation by DOX treatment and basal BAK expression suppression in *TP53*-KO cells (Fig. 4B), indicating that their expression is regulated by p53. However, introducing shRNA against *BAK* or *BAX* into A549 cells revealed no obvious delay in cell death (Fig. 4C, D). DOX treatment slightly suppressed LMP in *BAK*-knockdown (KD), but not *BAX*-KD cells (Fig. 4E). As BAK and BAX possess similar functions and work complementarily [27], cell death and LMP in *BAK/BAX* double knockdown (DKD) cells were assessed (Fig. 4F). Although DKD cell death was not delayed, cytoplasmic lysosomal enzyme leakage showed significant LMP suppression in DKD cells compared with control cells (Fig. 4G, H), though it was almost similar to that in *BAK-*KD cells. These data suggest that BAK and BAX are partially involved in DOX-induced LMP.

### TP53-dependent BID activation induces LMP

**Fig. 5:**
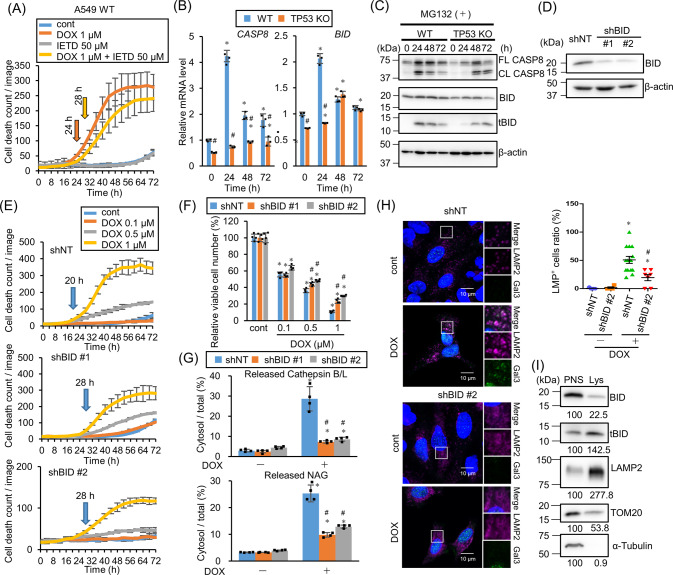
p53-dependent BID activation induces lysosomal membrane permeabilization (LMP). **A** A549 cells were treated with doxorubicin (DOX) in the presence of 50 µM z-IETD-fmk and the dead cell count was monitored every 4 h by live-cell imaging system with propidium iodide (PI) staining. **B** The relative *caspase-8* and *BID* mRNA expression level in wild-type (WT) or *TP53*-knockout (KO) A549 cells treated with 1 µM DOX for 24, 48, or 72 h was assessed by real-time PCR. *n* = 3, bar = mean ± SD, *p < 0.05 vs. control treatment, #*p* < 0.05 vs. WT. **C** CASP8 and BID activation were assessed by western blotting. WT or *TP53*-KO A549 cells were treated with 1 µM DOX in the presence of 10 µM MG132 for 24, 48, or 72 h. β-actin was used as a loading control. **D** *BID* knockdown efficiency in shRNA against BID (shBID #1 and #2)-expressing A549 cells was confirmed by western blotting. β-actin was used as a control. **E** shNT-, shBID #1-, or #2-expressing A549 cells were treated with DOX, and the dead cell count was monitored every 4 h by a live-cell imaging system with PI staining. **F** The viable shNT-, shBID #1-, or #2-expressing A549 cell count 48 h after DOX treatment was compared. *n* = 4, bar = mean ± SD, *p < 0.05 vs. cont., #*p* < 0.05 vs. shNT. **G** LMP in shNT-, shBID #1-, or #2-expressing A549 cells treated with 1 µM DOX for 48 h were measured by assessing released cytosolic N-acetyl glucosaminidase (NAG) or cathepsin B/L activity. *n* = 4, bar = mean ± SD, *p < 0.05 vs. cont., #*p* < 0.05 vs. shNT. **H** Galectin-3 (Gal3) and LAMP2 colocalization was assessed by immunofluorescent staining and confocal microscopic observation. shNT- or shBID #2-expressing A549 cells were treated with 1 µM DOX for 48 h and then immunostained for Gal3 (green), LAMP2 (magenta), and nuclei (blue). Cells treated with dimethyl sulfoxide (DMSO) for 48 h were used as a control. Scale bar = 10 µm. The boxed area was enlarged in the side panels. LMP^+^ cell ratio was measured and summarized in right. Bar = mean ± SE, *p < 0.05 vs. DOX ( − ), #*p* < 0.05 vs. shNT. **I** Truncated BID (tBID) expression in the lysosome-enriched fraction was assessed by western blotting. TMEM192–mRFP–3×HA-expressing A549 cells were treated with 1 µM DOX and 10 µM MG132 for 24 h and then the lysosomes were collected with anti-HA magnetic beads. Post nuclear supernatant (PNS) and eluted lysosome-enriched fraction (Lys) were used for western blotting. LAMP2, TOM20, and α-tubulin were used as lysosome, mitochondria, and cytosol markers respectively. Numbers indicate the relative band intensity of each protein.

To investigate other potential mechanisms for LMP induction, the BID involvement was further assessed. BID is another BCL-2 family member and activates BAK and BAX to induce apoptosis after the cleavage by caspase-8 [28]. BID induces LMP independently of BAX and BAK in RH-35 cells after exposure to H_2_O_2_ or palmitate, and causes RH-35 cell apoptosis by cytoplasmic lysosomal chymotrypsin B leakage [29]. In the presence of z-IETD-fmk, a caspase-8 inhibitor, DOX-induced cell death was suppressed and delayed (Fig. 5A), suggesting BID involvement in the DOX-induced LMP. *Caspase-8* and *BID* gene expression were upregulated by DOX treatment but they were apparently attenuated in *TP53*-KO cells (Fig. 5B). Western blotting shows the suppression of basal caspase-8 and BID expression in *TP53*-KO cells as compared with WT cells (Fig. 5C). As the truncated BID (tBID) is degraded by the ubiquitin proteasome system and its half-life is 1.5 h [30], MG132, a proteasome inhibitor, was co-administrated to block tBID degradation for tBID detection. caspase-8 and BID activation and cleavage were delayed in *TP53*-KO cells (Fig. 5C), indicating that caspase-8 and BID are expressed and activated *TP53*-dependently. Next, *BID*-KD cells were established by introducing two different shRNAs (Fig. 5D). DOX-induced cell death was apparently delayed and suppressed in both *BID-*KD cells (Fig. 5E, F). In addition, cytoplasmic cathepsin B/L and NAG leakage indicated that 60–70% DOX-induced LMP was suppressed (Fig. 5G). Immunofluorescence results indicated that Gal3 and LAMP2 colocalization increased in response to DOX treatment in control, but not shBID-expressing, cells (Fig. 5H). These data showed that BID plays an important role for DOX-induced LMP induction p53-dependently. In addition to DOX-induced LMP, CBDCA- and ETP-induced LMP were also suppressed in *BID*-KD cells, although the delay in cell death was moderate (Fig. S2). These results were almost reproducible in another human lung cancer cell line H226 with WT *TP53* (Fig. S3b). Since the KD efficiency was not as high as that in A549 cells, cell death was not delayed. Taken these data together, LMP is induced in response to DNA damage via BID activation. After cleavage by caspase-8, tBID localizes on mitochondrial outer membrane and is oligomerized to form a micelle-like pore structure, which induces apoptosis. In addition, several reports have shown tBID localization on lysosomes [29, 31]. To confirm this, lysosomes were isolated and western blotting was performed (Fig. 5I). Lysosome isolation concentrated the lysosomal protein LAMP2, but not mitochondrial or cytosolic proteins TOM20 and α-tubulin. Interestingly, tBID, but not the full length BID, was enriched in the lysosomal fraction after DOX treatment, indicating tBID localization on lysosomes.

### LMP triggers cell death in response to DNA-damaging drugs

**Fig. 6:**
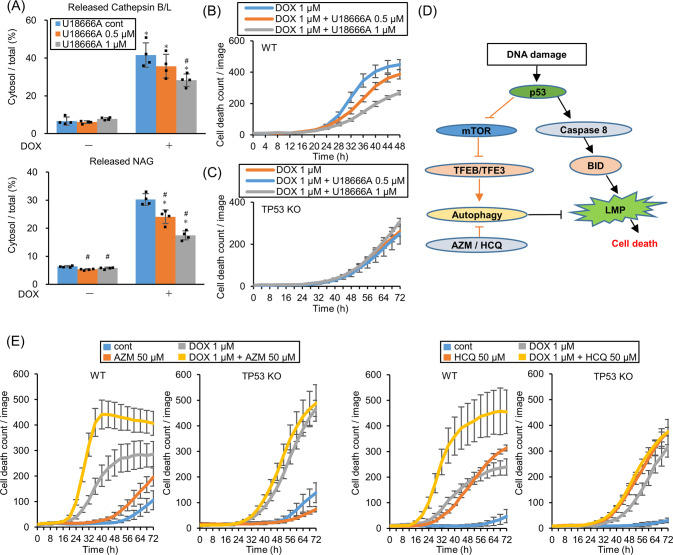
Stabilization of lysosomal membrane represses cell death whereas autophagy inhibition enhances cell death. **A** Lysosomal membrane permeabilization (LMP) in wild-type (WT) A549 cells treated with 0, 0.5, or 1 µM U18666A for 24 h before treating with 1 µM DOX for 48 h were measured by assessing released cytosolic N-acetyl glucosaminidase (NAG) or cathepsin B/L activity. *n* = 4, bar = mean ± SD, *p < 0.05 vs. DOX cont., #*p* < 0.05 vs. U18666A 0 µM. **B**, **C** WT and *TP53*-knockout (KO) A549 cells were treated with U18666A for 24 h before DOX treatment and dead cell count was monitored by a live-cell imaging system with propidium iodide (PI) staining. **D** The schema shows that p53 induces cell toxic LMP via the caspase-8–BID pathway and simultaneously induces cell-protective autophagy via the mTOR–TFEB/TFE3 pathway to suppress LMP. **E** WT and *TP53*-KO A549 cells were treated with 1 µM DOX and 50 µM azithromycin (AZM) or 50 µM hydroxychloroquine (HCQ) and dead cell count was monitored every 4 h by a live-cell imaging system with PI staining.

To investigate whether LMP is the direct executor for cell death in response to DNA-damaging drugs, LMP was suppressed using U18666 A, which inhibits Niemann-Pick C1 (NPC1), a cholesterol transporter localized on the lysosomal membrane. U18666A treatment prevents LMP by accumulating cholesterol on the lysosomal membrane to stabilize it in normal human fibroblasts [32]. As previously reported, cholesterol accumulates on lysosomes in A549 cells (Fig. S4). DOX and U18666A co-administration suppressed LMP and cell death in a U18666A dose-dependent manner (Fig. 6A, B). However, U18666A treatment did not suppress *TP53*-KO cell death (Fig. 6C). These data demonstrated that LMP itself triggers cell death in WT, but not *TP53*-KO, A549 cells. LMP was detected in *TP53*-KO cells after 72 h DOX treatment which is later than DOX-induced cell death initiation (Fig. 1), probably due to cell death-induced multi-organelle failure.

In addition to LMP induction, cytoprotective autophagy was simultaneously induced in response to DNA-damaging drugs via the p53–mTOR–TFEB pathway that suppressed LMP (Fig. 3). Hence, blocking autophagy should further enhance LMP-mediated cell death (Fig. 6D). To confirm this idea, DOX was co-administrated with autophagy inhibitors AZM or HCQ. DOX-induced cell death was pronounced in the presence of AZM or HCQ in WT, but not *TP53*-KO, A549 cells (Fig. 6E).

## Discussion

This study identifies the molecular mechanism of p53-mediated LMP induction in response to DNA-damaging drugs. LMP was executed by tBID via p53-mediated BID upregulation and activation by the cleavage by caspase-8, which is also upregulated by p53. Simultaneously, p53 also activated lysophagy to eliminate the damaged lysosomes by cytoprotective autophagy via TFEB/TFE3 (Fig. 6D). Although we confirmed BID-dependent LMP induction with H226 cells also, most experiments were performed with A549 cell line. To state the universality of this model, further experiment with other cell lines, including non-cancerous cell lines, would be needed. In addition, although p53-dependent AMPK activation was demonstrated, this study does not show the mechanism of p53-mediated AMPK activation. Sestrin, a p53 target gene and stress-induced metabolic protein, is involved in AMPK activation. The sestrin, activated by DNA damage, directly binds to AMPK and promotes AMPK autophosphorylation [33, 34]. Caspase-8 activation in WT cells was more pronounced than that in *TP53*-KO cells through an unknown mechanism (Fig. 5B, C). Previous reports have shown that expression of *FAS*, a death receptor gene, is regulated p53-dependently under DNA damage. In addition, FAS binds to Fas-associated cell death domain protein (FADD), an intracellular adaptor protein, after interaction with FAS-ligand, transmitting the cell death signal from FADD to caspase-8, thus activating caspase-8 [35]. In addition to the role of p53 as a transcription factor, a transcription-independent role for cell death has been established. Transcriptionally inactive p53 acts in cytosol or mitochondria to activate the apoptotic pathway through interacting with Bcl-2 family members [36]. Further study of our model is needed to determine whether LMP and autophagy induction by p53 depends on transcriptional activity.

In response to DNA-damaging drugs, WT A549 cells exhibited cell death earlier than *TP53-KO* A549 cells (Fig. 1). This early phase of cell death was mediated through LMP induction because LMP inhibition by caspase-8 inhibitor, *BID* KD, as well as exposure to U18666A for the lysosomal membrane stabilization, repress cell death execution in the early phase. However, LMP was still detected in *TP53*-KO cells by Gal3 puncta assay and lysosomal enzyme leakage after prolonged DOX exposure (Fig. 1D, F). Moreover, a previous study has demonstrated that DOX treatment induces apoptosis in *TP53*-KO as well as WT A549 cells [19]. Thus, LMP induction in *TP53*-KO cells detected after prolonged exposure to DNA-damaging drugs appears to be due to cell death execution, which causes multiple-organelle failure. Whether the LMP induction in the early phase affects drug sensitivity and therapeutic outcomes in cancer patients remains unknown. However, since autophagy inhibition reinforces cell death induction, autophagy induction in response to lysosomal damage appears to attenuate drug sensitivity. In addition, our findings provide important insight when we treat cancer patients with DNA-damaging drugs in combination with autophagy inhibitors.

In this study, LMP induction in response to DNA-damaging drugs was enhanced in *TFEB/TFE3* DKO A549 cells compared with WT A549 cells (Fig. 2D). Since TFEB/TFE3 are the master regulators of lysosome/autophagosome biogenesis, this enhanced LMP was probably due to the inhibition of the clearance of the damaged lysosomes by lysophagy. Indeed, LMP induction in response to DNA-damaging drugs was enhanced in autophagy-deficient *ATG5*-KO A549 cells as well as in the presence of autophagy inhibitors HCQ and AZM (Figs. 3E, 6E). These data indicate that the p53–mTOR–TFEB/TFE3 axis represses LMP via lysophagy induction. However, TFEB/TFE3 amplifies LMP in response to ETP in mouse embryonic fibroblasts (MEFs) [21]. This discrepancy may be due to the type of the cells used for the experiments, namely cancer cell lines and normal MEFs. In addition, TFEB/TFE3 stabilizes p53 by suppressing E3 ligase MDM2, thereby suppressing p53 activation in *TFEB/TFE3* DKO cells [21]. Our study indicates that p53 exhibited dichotomous nature for LMP; induction via tBID and repression via TEEB/TFE3-mediated lysophagy induction. Thus, in TFEB/TFE3 DKO cells, DNA damage failed to induce not only autophagy due to the lack of TFEB/TFE3, but also LMP due to p53–BID pathway suppression by p53 de-stabilization. In this context, LMP execution initiated by p53 may be determined by the fine tuning between p53–mTOR–TFEB/TFE3 and p53–tBID pathways. Autophagy, a self-recycling system, is upregulated in cancer cells than that in normal cells [37]. Autophagy dependency may be higher in A549 cells than that in MEFs, which may cause the different outcomes of LMP induction under p53 control.

BID, a pro-apoptotic Bcl-2 family member, localizes in the cytoplasm. However, when TNFα or FAS signaling pathway is activated, caspase-8 cleaves BID to form the tBID, which translocates to mitochondria; subsequently, tBID activates other pro-apoptotic Bcl-2 family members BAK or BAX to form the mitochondrial membrane pore [30]. Although several reports show that BAK and BAX induce LMP [26, 38], these molecules were partially involved in the p53-induced LMP in this study. This study demonstrated that BID induces LMP probably independent from the BAK/BAX because *BID* KD much strongly repressed LMP than *BAX/BAK* DKD. Supporting these findings, another study has reported that tBID, but not BAK/BAX, punctures lysosomes in vitro using a model membrane that mimics the lipid structure of lysosomes [31]. As this study reports tBID localization on isolated lysosomes, tBID may directly induce LMP. In a previous study, tBID monomers were inserted into the lipid bilayer, which then combine to form tBID dimers. Further oligomerization formed higher-order tBID oligomers [39]. Large oligomers have a transmembrane structure and enhance membrane permeability [40]. The oligomerized tBID may form pores on lysosomes similar to those on mitochondria, and induce LMP.

This study showed that p53 activation by DNA damage simultaneously induces LMP-mediated cell death and cytoprotective autophagy. This response might be also involved in cellular senescence. In senescent cells, LMP is induced and H^+^ is leaked from the damaged lysosomes to reduce the intracellular pH [41], whereas the activation of lysosomal enzymes, such as senescence-associated beta-galactosidase (SA-β gal), and lysosome count increment induce cellular senescence [42, 43]. TFEB/TFE3-dependent lysosomal biogenesis and BID-dependent LMP seem to occur simultaneously in senescent cells as observed in this study. Thus, p53-dependent LMP and autophagy regulation might be involved in many aspects of cellular biology.

## Materials and methods

### Reagents

DOX hydrochloride, HCQ sulfate, and LLOMe were purchased from Cayman Chemical (Ann Arbor, MI, USA). ETP (Lastet Injection) was purchased from Nippon Kayaku (Tokyo, Japan). CBDCA (carboplatin injection) was purchased from NICHI-IKO (Toyama, Japan). AZM were purchased from Tokyo Chemical Industry (Tokyo, Japan). Baf was purchased from Wako (Osaka, Japan). MG132 was purchased from Peptide Institute (Osaka, Japan). U18666A was purchased from Sigma-Aldrich (St. Louis, MO, USA). Z-IETD-FMK was purchased from Selleck Chemicals (Houston, TX, USA).

### Cell lines and culture conditions

Human lung adenocarcinoma-derived A549 and H226 cells were obtained from the American Type Culture Collection (ATCC; Manassas, VA, USA) and cultured in Dulbecco’s modified Eagle medium supplemented with 10% fetal bovine serum and 1% penicillin/streptomycin at 37 °C in a humidified incubator containing 5% CO_2_ and 95% air. All experiments were conducted within 10 passages after thawing. Mycoplasma contamination was tested routinely using the e-Myco^TM^ Mycoplasma PCR Detection kit ver.2.0 (iNtRON Biotechnology, Inc.; Seongnam, Korea).

### Establishment of KO cell lines

To establish the KO cells, the CRISPR–Cas9 system was used. A549 cells were transfected with pSpCas9 (BB)-2A-Puro (PX459) V2.0 plasmid vector (gifted by Dr. Feng Zhang; plasmid cat. no. 48139; Addgene) [44] containing a guide sequence for TP53, TFEB, TFE3, or ATG5. Thereafter, the cells were selected with puromycin for 2 days, and grown without puromycin to select single colonies. The guide RNA sequences are summarized in Table. S1.

### Lentiviral production and gene KD or expression

Lentiviruses were produced in HEK293T cells by transfection with the following plasmids: pMD2.G (Addgene #12259, gifted by Didier Trono), psPAX2 (Addgene #12260, gifted by Didier Trono), and pLKO.1 shRNA expression vector (Addgene #8453, gifted by Bob Weinberg) [45], pLJM1–Tmem192–mRFP–3xHA vector (Addgene #134631, gifted by Roberto Zoncu) [46], or pLenti–AcGFP–Gal3. *BAX*, *BAK*, and *BID*-targeting shRNA vectors and shNT non-targeting control vector were constructed using the sequences shown in Table S1. A549 cells were infected with viruses and subjected to puromycin or blasticidin selection to isolate knockdown or overexpressing cells.

### Assessment of cell death

Cell death was assessed by staining with PI (Wako Pure Chemicals Corporation) and counting the red fluorescent signals every 4 h using the IncuCyte ZOOM automated live-cell imaging system (Sartorius; Goettingen, Germany). The cells were treated with DOX, ETP, or CBDCA in the presence of 2.5 µg/mL PI for 72 h in 96-well plates in quadruplicate [47].

The cell death initiation point was defined as follows: the highest and lowest dead cell count under drug treatment was defined as 100 and 0, respectively. Cell death onset was defined as the point where the difference between the control and drug-treated dead cell counts was >6.

### Cell viability assay

Cellular viability was assessed using the CellTiter Blue Cell Viability Assay kit (Promega; Madison, WI, USA) according to the manufacturer’s instructions. Briefly, 6 × 10^3^ cells/well were seeded to a 96-well flat-bottomed culture plate and pre-cultured overnight. Once fully adhered to the plate, the medium was replaced, and the cells were treated with 0.1–1 µM DOX for 48 h. All untreated controls were supplemented with dimethyl sulfoxide (DMSO) to match the DOX treatment volume. During the last 1.5 h, CellTiter Blue reagent was added to each well, and fluorescence was measured (excitation, 560 nm; emission, 590 nm) using a SpectraMax iD3 fluorometer (Molecular Devices LLC; San Jose, CA, USA). The mean fluorescence relative to that of the untreated cells was expressed as a percentage of cellular viability.

### Western blotting

Cells were lysed with RIPA lysis buffer (Nacalai Tesque; Kyoto, Japan) supplemented with a protease and phosphatase inhibitor cocktail (Nacalai Tesque). Equal amounts of proteins were loaded on the gels, separated by sodium dodecyl sulfate–polyacrylamide gel electrophoresis (SDS–PAGE), and transferred to Immobilon-P membranes (Millipore Corp.; Bedford, MA, USA). These membranes were probed with primary antibodies, such as anti-p53 antibody (Ab) (sc-126, 1:1,000), anti-β-actin Ab (sc-47778, 1:1,000), anti-LAMP1 Ab (sc-20011, 1:1000), anti-LAMP2 Ab (sc-18822, 1:1000), anti-TOM20 (FL-145) Ab (sc-11415, 1:1000), and anti-Bax (N-20) Ab (sc-493, 1:1000) (all purchased from Santa Cruz Biotechnology; Santa Cruz, CA, USA); anti-microtubule-associated protein 1 light chain 3B (LC3B) Ab (NB600-1384, 1:4000) (purchased from Novus Biologicals, Inc.; Littleton, CO, USA); anti-phospho-AMPKα (Thr172) (40H9) Ab (#2535, 1:1000), anti-AMPKα (D63G4) Ab (#5832, 1:1000), anti-phospho-S6 ribosomal protein (Ser235/236) (D57.2.2E) XP® Ab (#4858, 1:1000), anti-S6 ribosomal protein (5G10) Ab (#2217, 1:1000), anti-TFEB Ab (#4240, 1:1000), anti-caspase-8 Ab (#9746 S, 1:1000), anti-ATG5 Ab (#12994 S, 1:1000), anti-BID Ab (#2002, 1:1000), and anti-α-Tubulin Ab (#3873, 1:1000) (Cell Signaling Technology; Danvers, MA, USA); anti-TFE3 Ab (HPA023881, 1:1000) and anti-Bak Ab (06-536, 1:1000) (both purchased from Sigma-Aldrich). Uncropped original images merged with corresponding marker images are shown in Figs. S5–11.

### Immunofluorescence staining and confocal microscopic observation

To evaluate the Gal3 puncta, immunofluorescence was performed. Cells were seeded on 13 mm glass coverslips in a 12-well culture plate, and then treated with DMSO and DOX for 48 h. Coverslips were washed twice with phosphate-buffered saline (PBS), and then fixed for 10 min with ice-cold methanol at −20 °C. After washing twice with PBS and once with Tris-buffered saline with Tween 20 (TBST), the coverslips were exposed to TBST containing 10% normal goat serum (Vector Laboratories; Burlingame, CA, USA) for 1 h at room temperature to block non-specific binding. The cells were incubated with primary antibodies such as anti-Gal3 Ab (#87985 S, 1:200) and anti-LAMP2 Ab (sc-18822, 1:100) (Santa Cruz Biotechnology) at 4 °C overnight. The coverslips were washed three times with TBST at room temperature, and subsequently incubated with Alexa Fluor® 488-conjugated goat anti-rabbit IgG (H + L) or Alexa Fluor® 555-conjugated goat anti-mouse IgG (H + L) secondary Ab (1:250; Thermo Fisher Scientific; Waltham, MA USA) for 1 h at room temperature. The coverslips were washed with TBST, and mounted in ProLong® Diamond Antifade Mountant (Thermo Fisher Scientific). Nuclei were stained with 4′,6-diamidino-2-phenylindole (DAPI; 1:1000, #D9542; Sigma), and the cells were imaged using a confocal laser scanning fluorescence microscope (LSM 700, Carl Zeiss, Germany). DMSO-treated cells were used as a negative control. Cells with five or more Gal3 puncta were defined as LMP-positive cells. The LMP-positive cell percentage per field was calculated for multiple fields. To confirm AcGFP–Gal3 localization on lysosomes, anti-GFP Ab (012-20461, 1:200; Wako), anti-LAMP2 Ab (sc-18822, 1:100; Santa Cruz Biotechnology), Alexa Fluor® 488-conjugated goat anti-mouse IgG2a, and Alexa Fluor® 555-conjugated goat anti-mouse IgG1 secondary antibodies (1:250; Thermo Fisher Scientific) were used.

### LMP measurement

AcGFP–Gal3-expressing A549 cells were observed using a confocal laser scanning fluorescence microscope (LSM 700) without fixation and the number of Gal3 puncta in each cell was counted to measure the LMP. Cells with five or more Gal3 puncta were defined as LMP-positive cells. The LMP-positive cell percentage per field was counted in multiple fields.

Alternatively, the cytosolic and total enzymatic activities of cathepsin and β-N-acetyl glucosaminidase (NAG) were assessed to calculate LMP, as described in previous reports [19]. Briefly, 6 × 10^4^ cells/well were seeded in a 24-well plate and then treated with DOX for 24, 48, or 72 h, CBDCA for 66 h, or ETP for 29 h. After washing with PBS, the cells were solubilized with 15 (for cytosol) or 200 µg/mL (for total) digitonin in DE buffer (250 mM sucrose, 20 mM HEPES, 10 mM KCl, 1.5 mM MgCl_2_, 1 mM EDTA, 1 mM EGTA, pH 7.5, with 0.5 mM Pefabloc). The lysates were used for cathepsin or NAG activity assay and measuring protein concentration with a BCA kit (Thermo). Cathepsin activity was measured in cathepsin reaction buffer (50 mM sodium acetate, 4 mM EDTA, pH 6.0) supplemented with 0.5 mM Pefabloc, 8 mM dithiothreitol, and 50 µM z-FR-AMC (Peptide Institute) by assessing the fluorescent intensity (Ex 400/Em 489 nm) every minute for 30 min at 30 °C. For NAG activity assay, the lysate was mixed with NAG reaction buffer (0.2 M sodium citrate buffer, 300 µg/mL 4-methylumbelliferyl N-acetyl-β-D-glucosaminide pH 4.5) and incubated at 37 °C for 30 min. Then, Tris buffer (0.5 M Tris-HCl, pH 10.4) was added, and the fluorescence (Ex 360/ Em 440 nm) was measured. Each enzymatic activity was standardized according to protein concentration, and the cytosol/total cathepsin or NAG activity was calculated. All experiments were performed in six replicates for each condition, and statistical analysis was performed after excluding the highest and lowest values at each point.

### Lysosome immunoprecipitation

For lysosome immunoprecipitation experiments, T192–mRFP–3×HA-expressing A549 cells were lysed and intact lysosomes were immunoprecipitated using anti-HA-conjugated Dynabeads (Thermo Scientific, #88837) as reported previously [48]. Briefly, 6 × 10^6^ cells/well were seeded in a 15 cm dish. The next day, the cells were pre-treated with MG132 for 2 h and then treated with DOX for 24 h. The treated cells were collected and washed twice with ice-cold KPBS buffer (136 mM KCl and 10 mM KH_2_PO_4_, pH 7.25), pelleted, resuspended in 1 mL KPBS supplemented with protease inhibitor cocktail and 50 mM sucrose, homogenized with Dounce homogenizer, and centrifuged at 2700 *rpm* for 10 min. The supernatant was harvested and incubated with 50 µL anti-HA magnetic beads with rotation for 20 min. The beads were washed twice with 150 mM NaCl-supplemented KPBS and once with KPBS. The immunoprecipitated lysosomes were eluted from beads with 0.1% NP-40-supplemented KPBS at 37 °C for 30 min.

### Real-time PCR

To assess the mRNA expression level, cDNA was synthesized using PrimeScript RT Master Mix (Takara Bio, Inc.; Otsu, Japan) with total RNA extracted from A549 cells using a NucleoSpin RNA kit (Takara Bio, Inc.). Gene expression level was determined by qPCR using TB Green Premix Ex Taq II (Tli RNaseH Plus)(Takara Bio, Inc.). Changes in target mRNA expression were calculated using the Δ(ΔC_T_) method. HPRT was used as an internal control. Primer sequences were obtained from Harvard Primer Bank [49–51] and are listed in Table S1.

### Statistical analysis

All quantitative data are expressed as mean ± standard deviation (SD). Statistical analyses of cell death assay and qPCR were performed with two-way analysis of variance (ANOVA), followed by Tukey’s multiple comparison test. For all other assays, one-way ANOVA, followed by Tukey’s multiple comparison test was used. Statistical significance was set at *p* < 0.05. Variation across experimental groups was analyzed using *F*-testing. All analyses were performed using the IBM SPSS Statistics (IBM).

## Supplementary information

Supplemental figures and table

Reproducibility checklist

## Data Availability

The datasets generated during and/or analyzed during the current study are available from the corresponding author on reasonable request.
