# Supplementary material for: p53 regulates lysosomal membrane permeabilization as well as cytoprotective autophagy in response to DNA-damaging drugs
Source: Cell Death Discov. 2022 Dec 29;8:502. doi: 10.1038/s41420-022-01293-x (PMC9800408; doi:10.1038/s41420-022-01293-x)
Supplement: Supplementary file 1 — Supplemental figures and table [file 41420_2022_1293_MOESM1_ESM.pdf]

p53 regulates lysosomal membrane permeabilization as well as cytoprotective autophagy in response to DNA-damaging drugs

Gai Yamashita, Naoharu Takano, Hiromi Kazama, Kiyoaki Tsukahara and Keisuke Miyazawa

List of the materials included

Supplemental Figure 1. Aequorea coerulescens green fluorescence protein-conjugated galectin-3 (AcGFP–Gal3) co-localizes with LAMP2 during lysosomal membrane permeabilization (LMP) induction.

Supplemental Figure 2. *BID* knockdown in A549 cells suppresses lysosomal membrane permeabilization (LMP) in response to DNA damage.

Supplemental Figure 3. Lysosomal membrane permeabilization (LMP) and cell death are suppressed by *BID* knockdown (KD) in H226 cells.

Supplemental Figure 4. U18666A accumulates cholesterol in lysosomes.

Supplemental Figure 5-11. Original immunoblot images merged with corresponding marker images.

Supplemental Table 1. Oligo DNA sequences used for gene knockout, shRNA vector construction, and real-time PCR.

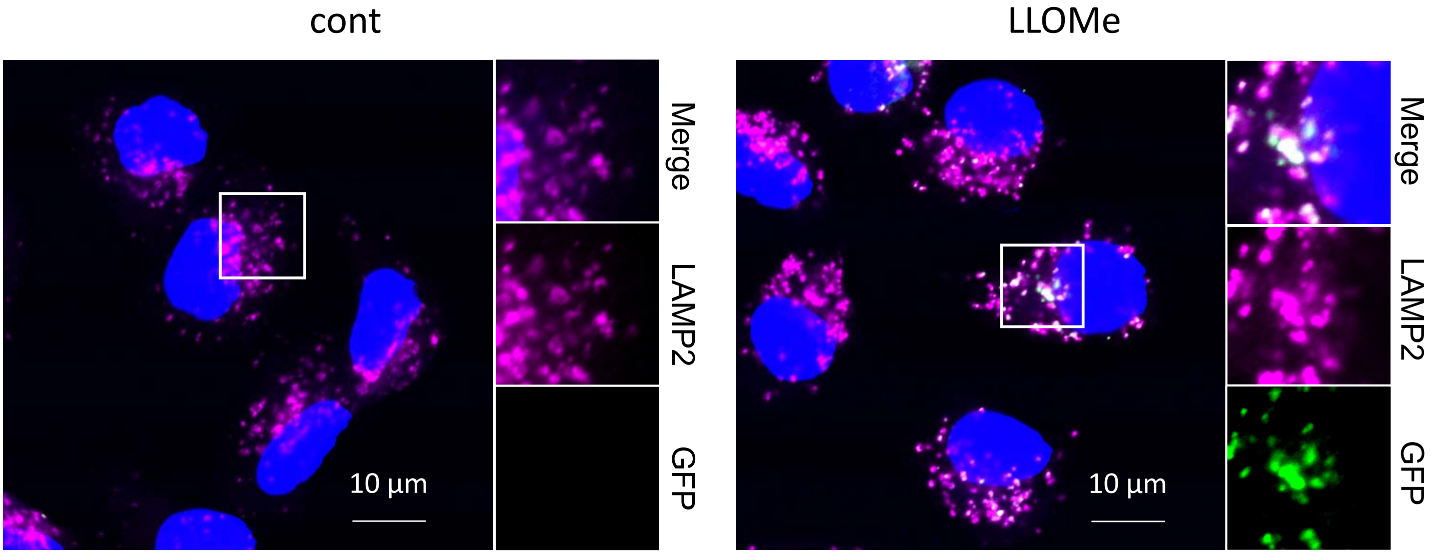

Supplemental Figure 1. Aequorea coerulescens green fluorescence protein-conjugated galectin-3 (AcGFP–Gal3) co-localizes with LAMP2 during lysosomal membrane permeabilization (LMP) induction. AcGFP–Gal3 and LAMP2 localization was assessed by immunofluorescent staining and confocal microscopic observation. AcGFP–Gal3-expressing A549 cells were treated with 1 mM L-Leucyl-L-Leucine methyl ester (LLOMe) for 4 h and then immunostained for green fluorescence protein (GFP, green), LAMP2 (magenta), and nuclei (blue). The boxed area was enlarged in the side panels. The cells treated with dimethyl sulfoxide (DMSO) for 4 h were used as a control. Scale bar=10 µm.

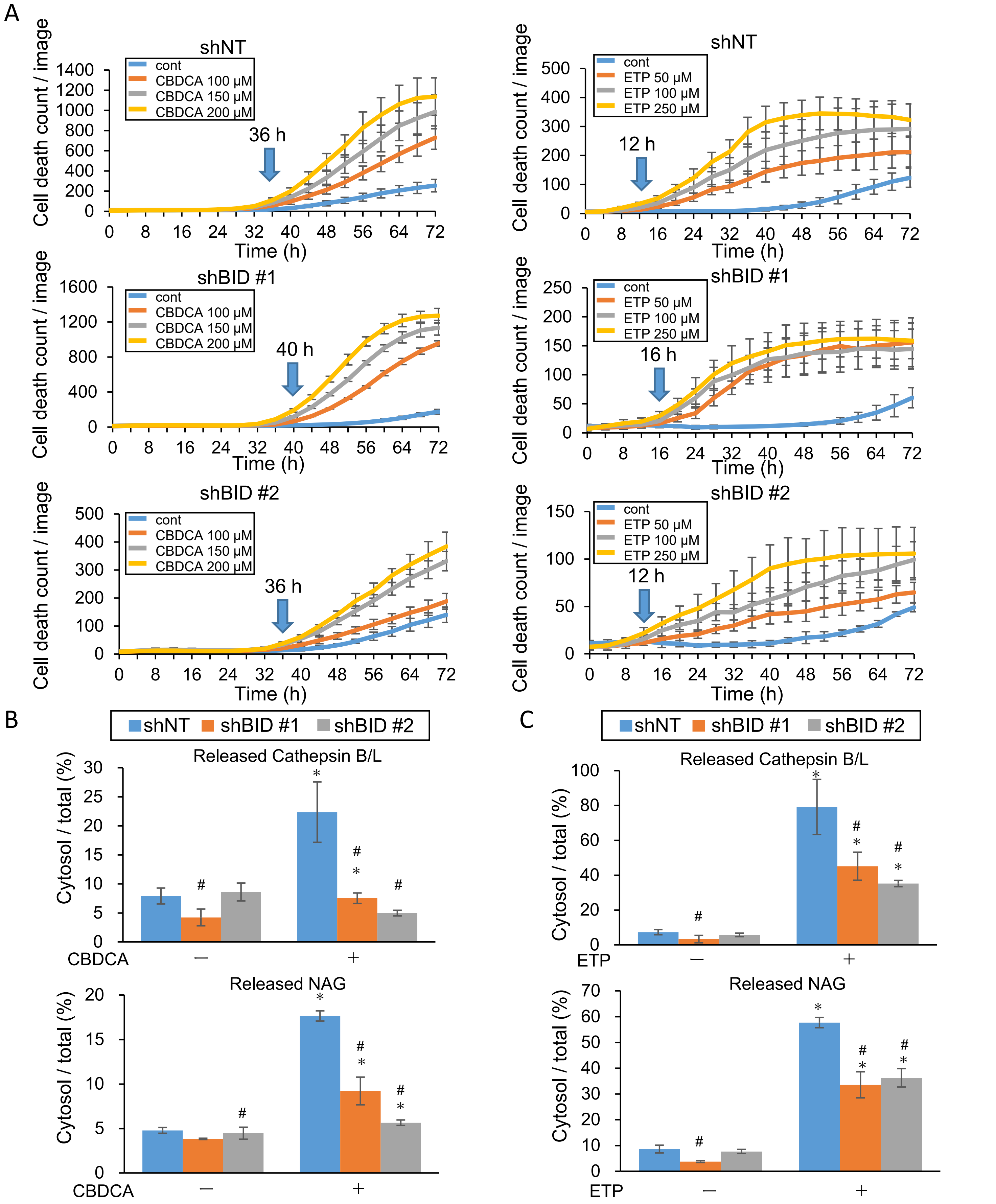

Supplemental Figure 2. *BID* knockdown in A549 cells suppresses lysosomal membrane permeabilization (LMP) in response to DNA damage. A, shNT-, shBID #1-, or #2-expressing A549 cells were treated with etoposide (ETP) or carboplatin (CBDCA) and dead cell count was monitored by a live-cell imaging system with propidium iodide (PI) staining. The cell death initiation point after 250 µM ETP or 200 µM CBDCA treatment is indicated by blue arrows. B, C, LMP in shNT-, shBID #1-, or #2-expressing A549 cells treated with (B) 150 µM CBDCA for 66 h or (C) 250 µM ETP for 29 h were measured by assessing released cytosolic N-acetyl-glucosaminidase (NAG) or cathepsin B/L activity. n=4, bar = mean ± SD, *p<0.05 vs. cont., #p<0.05 vs. shNT.

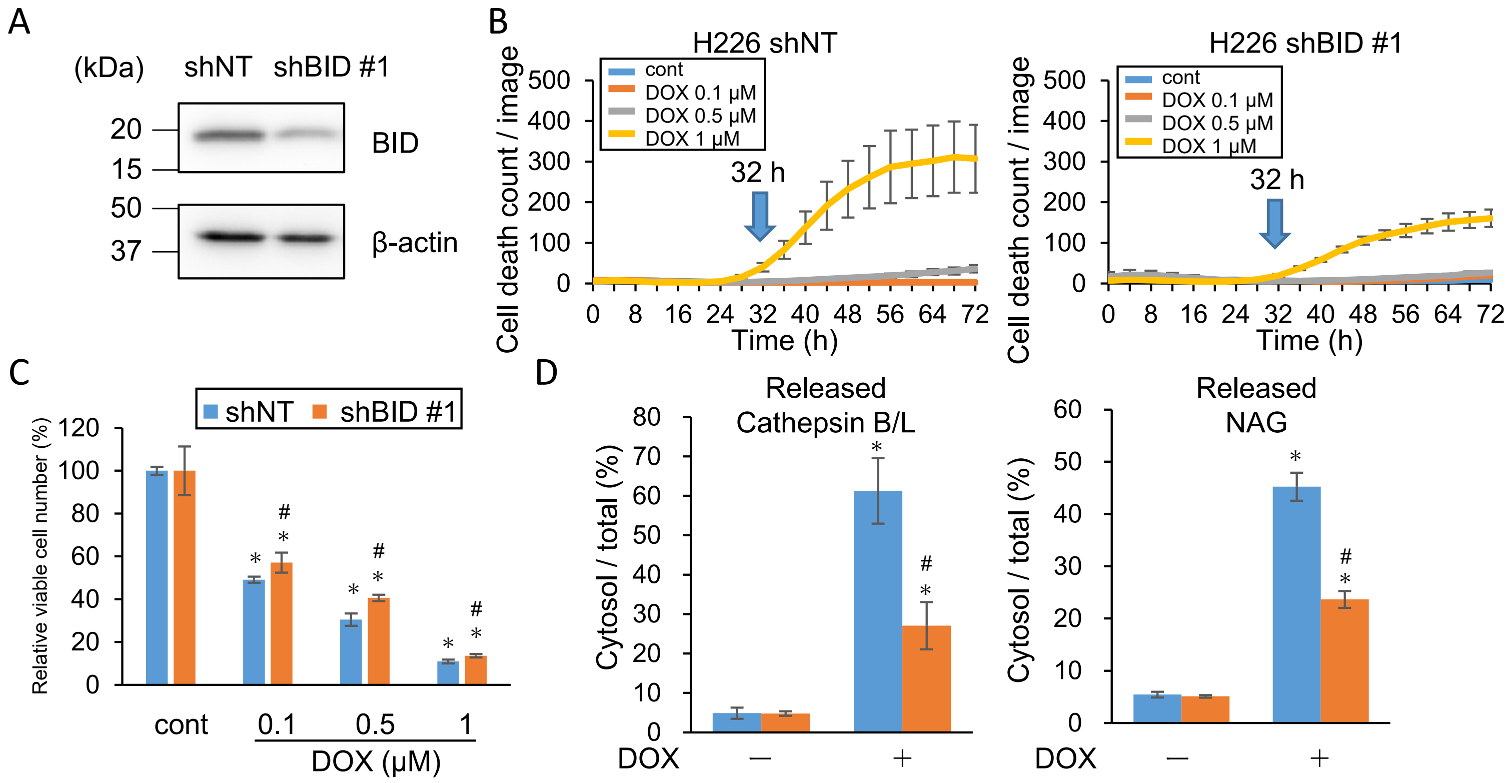

Supplemental Figure 3. Lysosomal membrane permeabilization (LMP) and cell death are suppressed by *BID* knockdown (KD) in H226 cells. A, *BID* KD efficiency in shRNA against BID (shBID #1)-expressing H226 cells was confirmed by western blotting. β-actin was used as a control. B, shNT- and shBID #1-expressing H226 cells were treated with doxorubicin (DOX) and the cell death was monitored using a live-cell imaging system with propidium iodide (PI) staining. The cell death initiation point after 1 μM DOX treatment is indicated by blue arrows. C, The viable cell count 48 h after DOX treatment was compared between shNT- and shBID #1-expressing H226 cells. n=4, bar = mean ± SD, *$p<0.05$ vs. cont., #$p<0.05$ vs. shNT. D, LMP in shNT- or shBID-expressing H226 cells treated with 1 μM DOX for 48 h were measured by assessing released cytosolic N-acetyl glucosaminidase (NAG) or cathepsin B/L activity. n=4, bar = mean ± SD, *$p<0.05$ vs. cont., #$p<0.05$ vs. shNT.

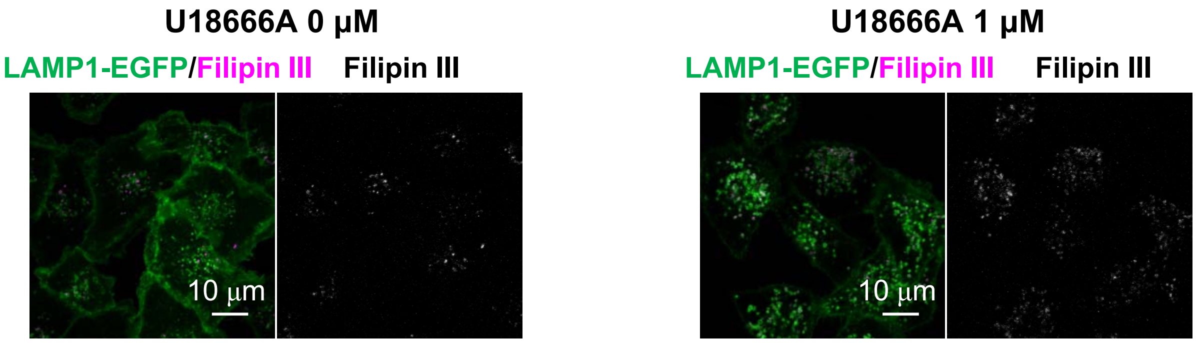

Supplemental Figure 4. U18666A accumulates cholesterol in lysosomes. Cholesterol accumulation in lysosomes is confirmed by confocal microscopic observation. LAMP1–EGFP-expressing A549 cells were treated with 1 µM U18666A for 24 h and then stained with 50 µg/mL Filipin III for 2 h. In the left panels, LAMP1 and Filipin III signals are shown in green and magenta, respectively. In the right panels, Filipin III signals are shown in white. The cells treated with dimethyl sulfoxide (DMSO) for 24 h were used as a control. Scale bar=10 µm.

Fig. 1A

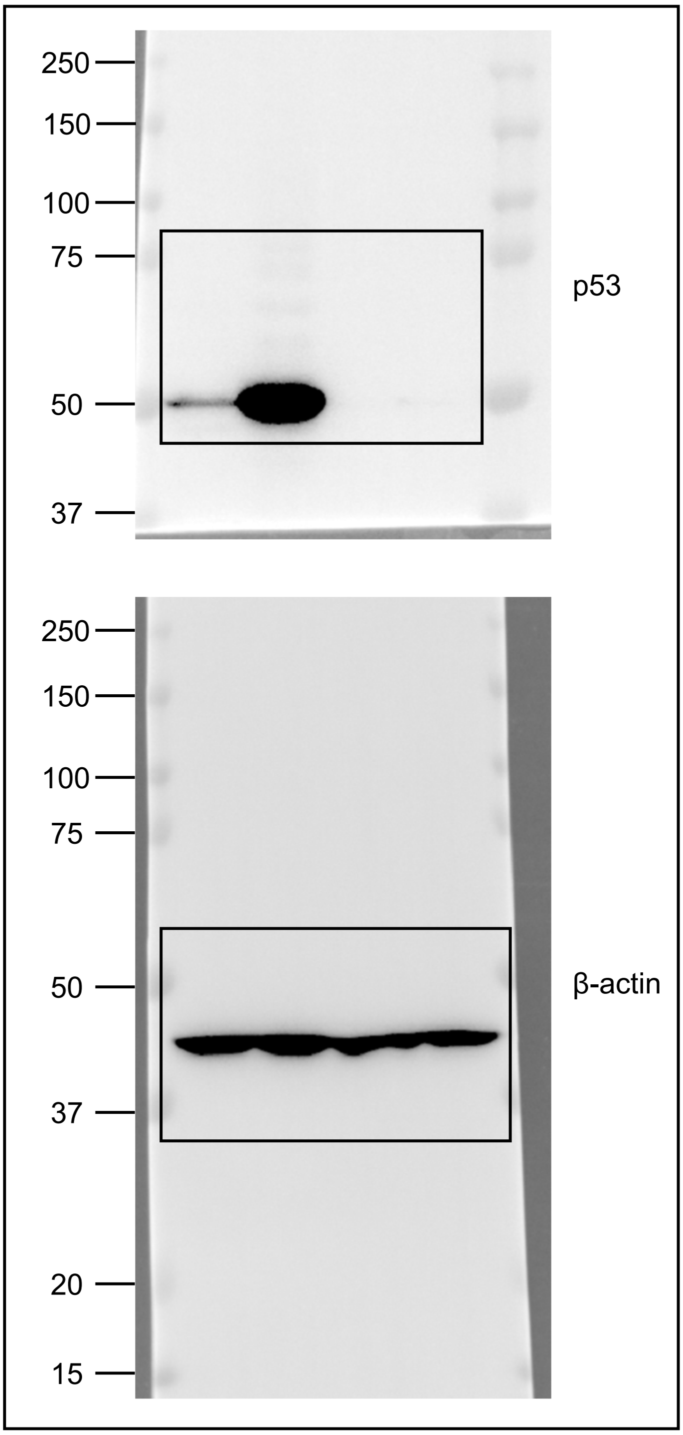

Supplemental Figure 5. Original immunoblot images merged with corresponding marker images.

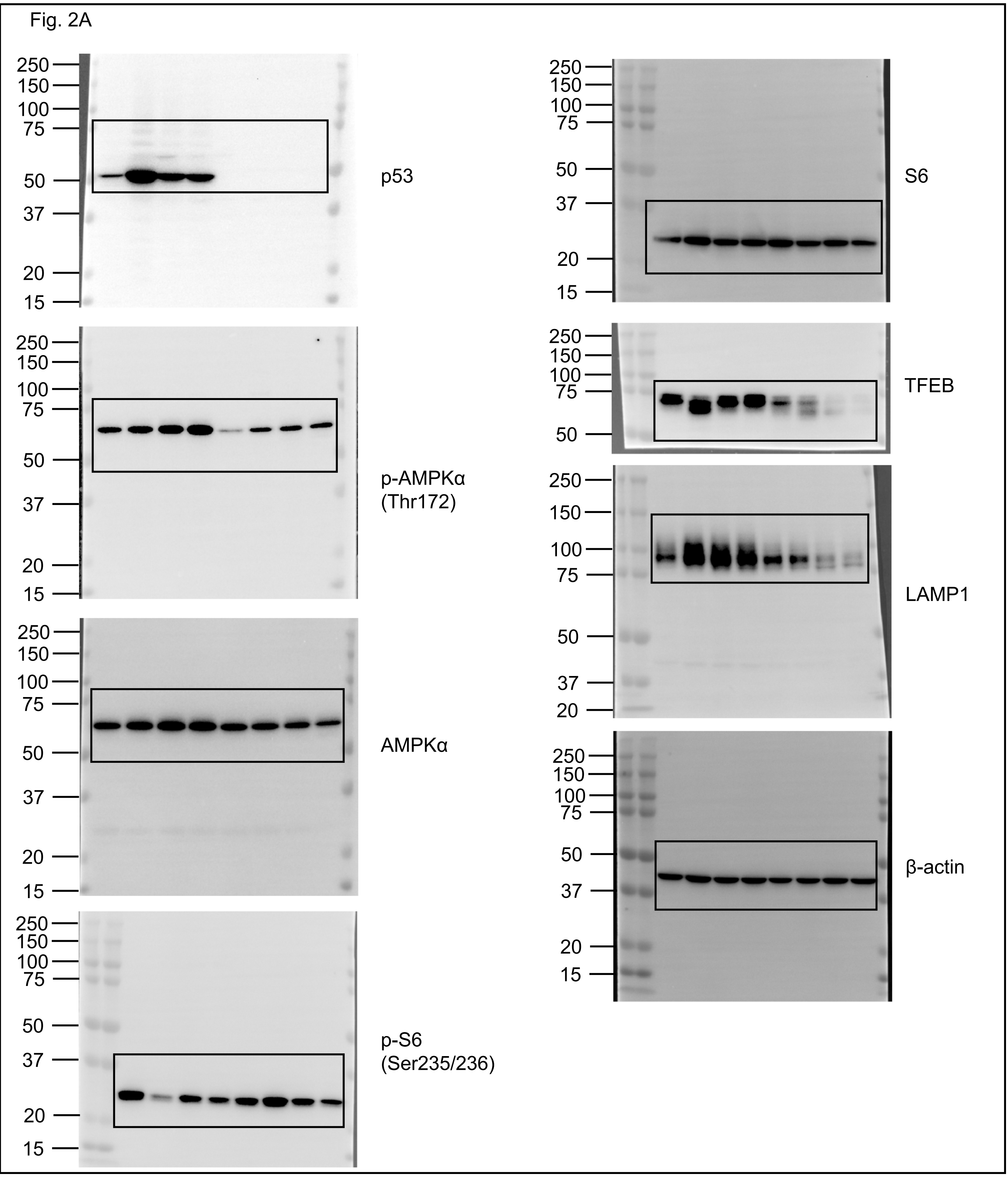

Supplemental Figure 6. Original immunoblot images merged with corresponding marker images.

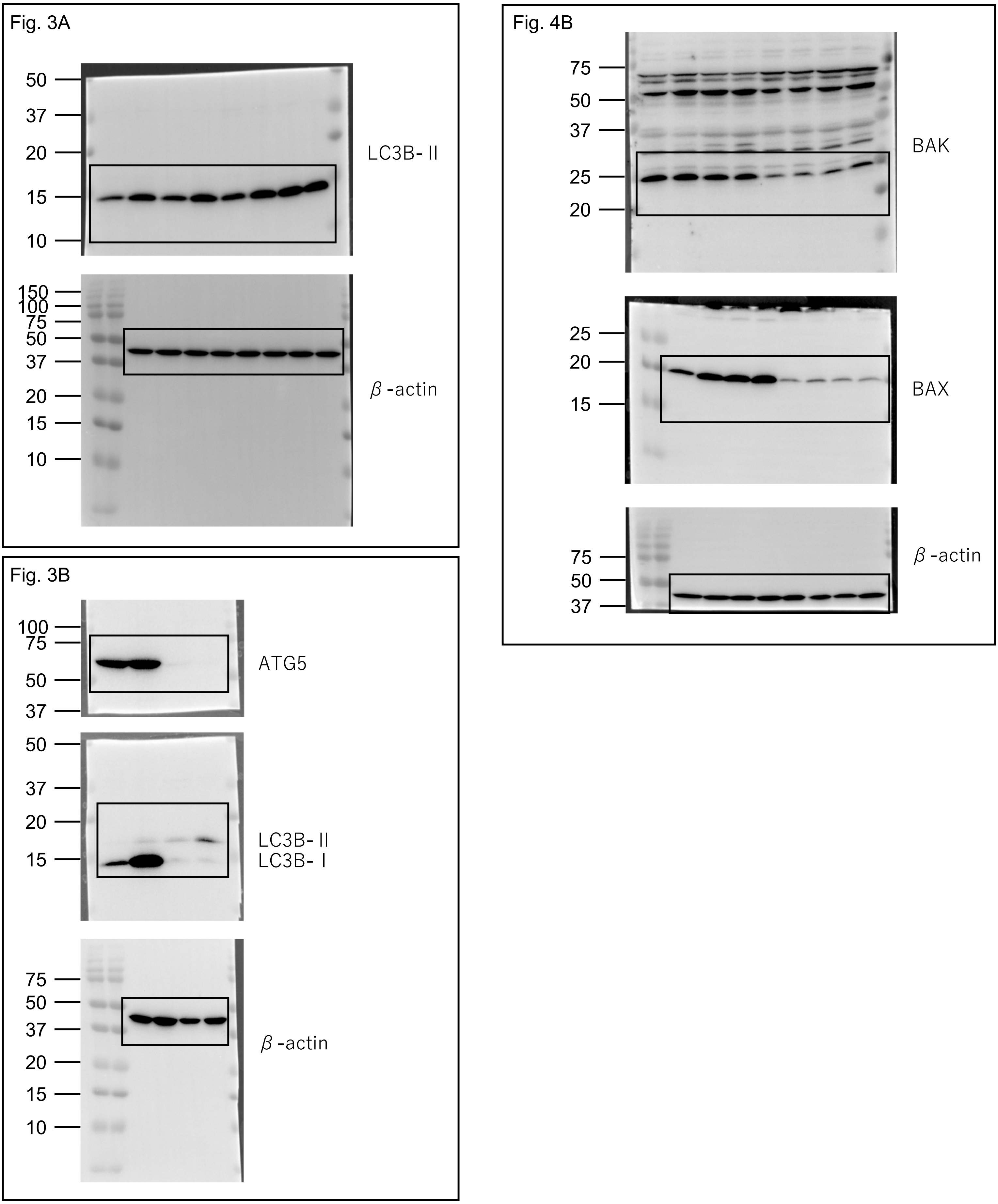

Supplemental Figure 7. Original immunoblot images merged with corresponding marker images.

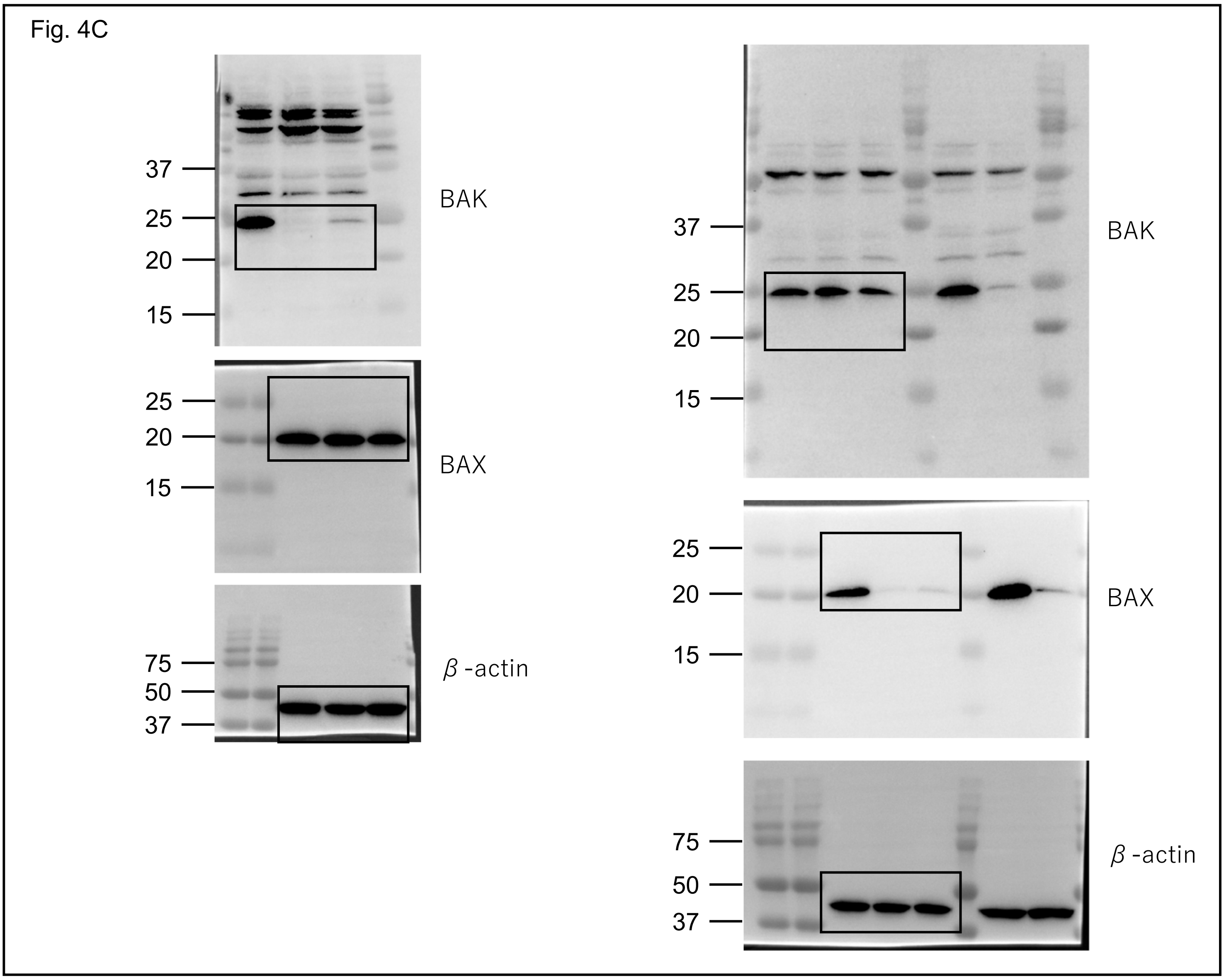

Supplemental Figure 8. Original immunoblot images merged with corresponding marker images.

Fig. 4F

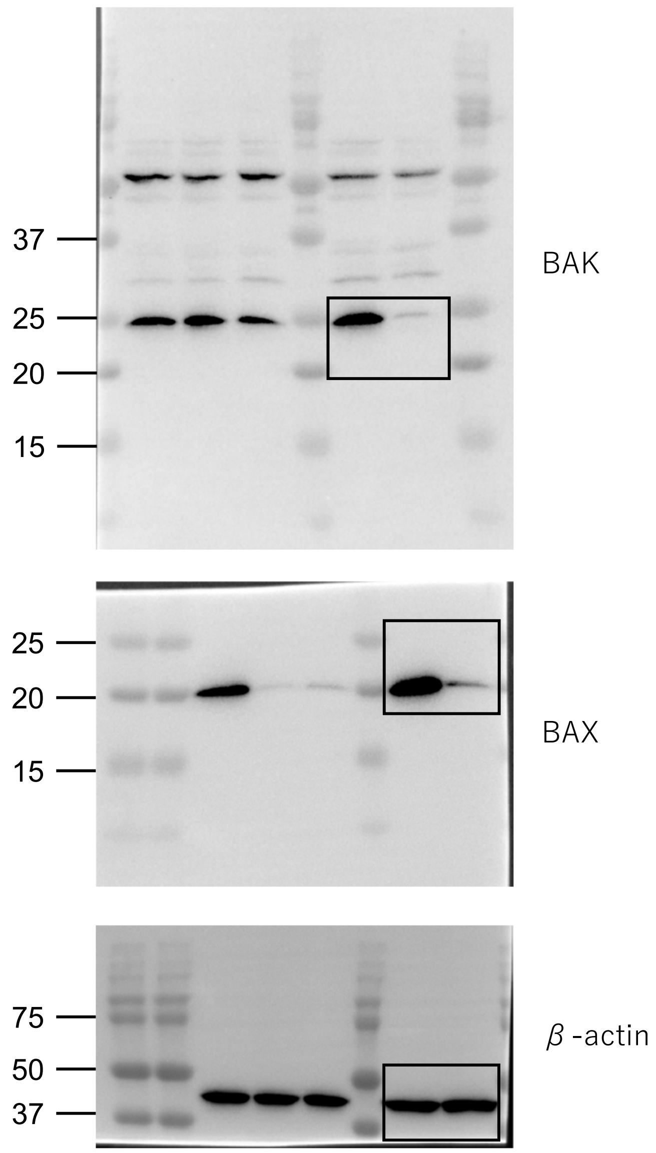

Supplemental Figure 9. Original immunoblot images merged with corresponding marker images.

Fig. 5C

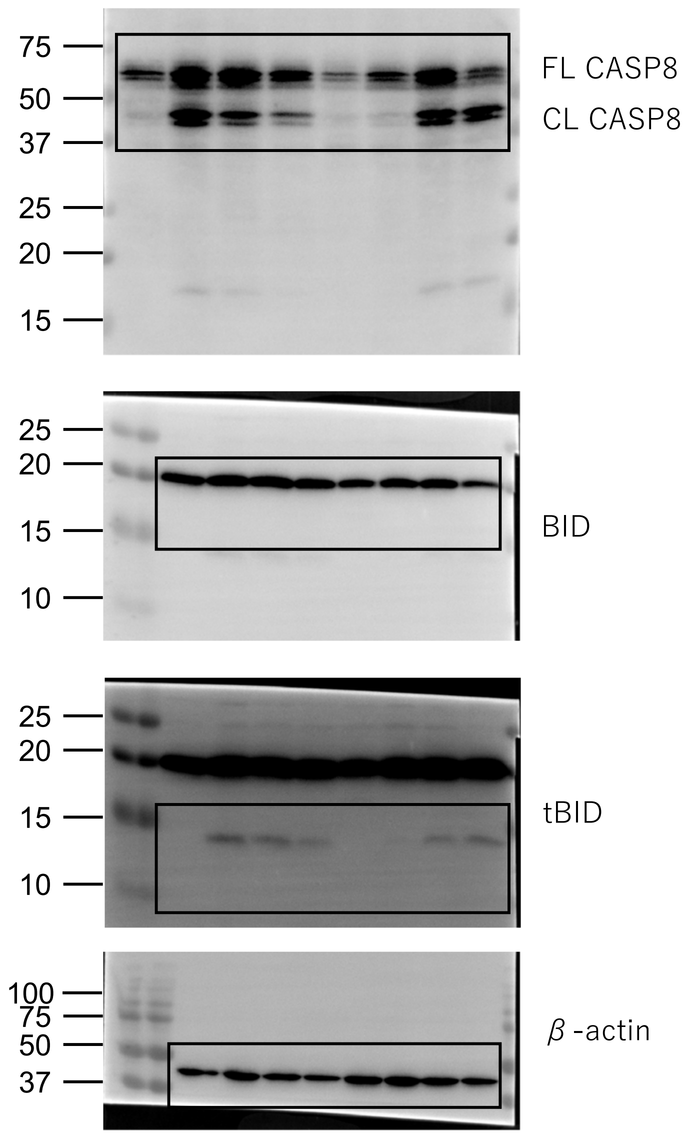

Fig. 5D

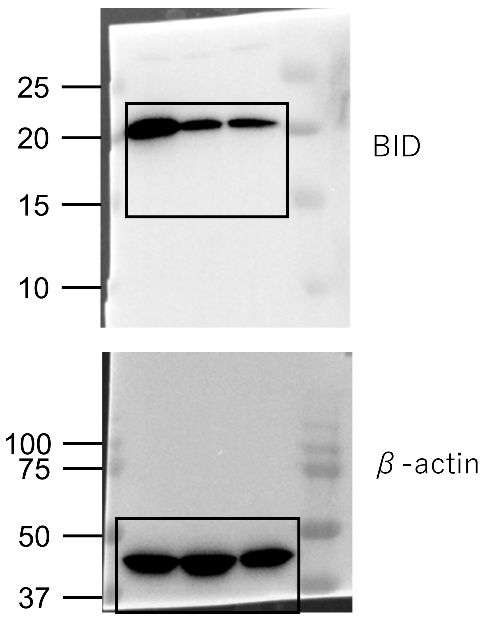

Supplemental Figure 10. Original immunoblot images merged with corresponding marker images.

Fig. 5I

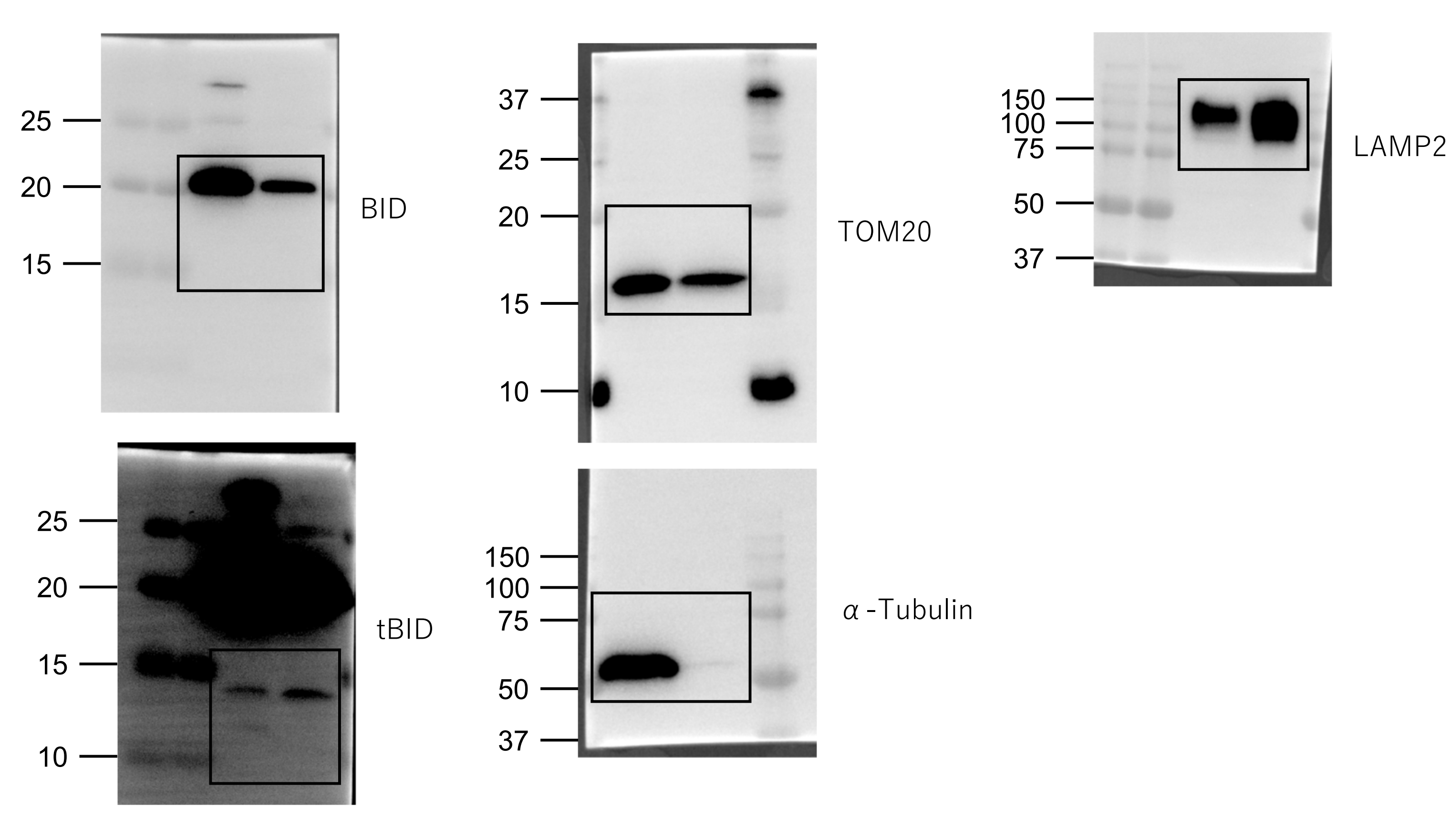

Fig. S3A

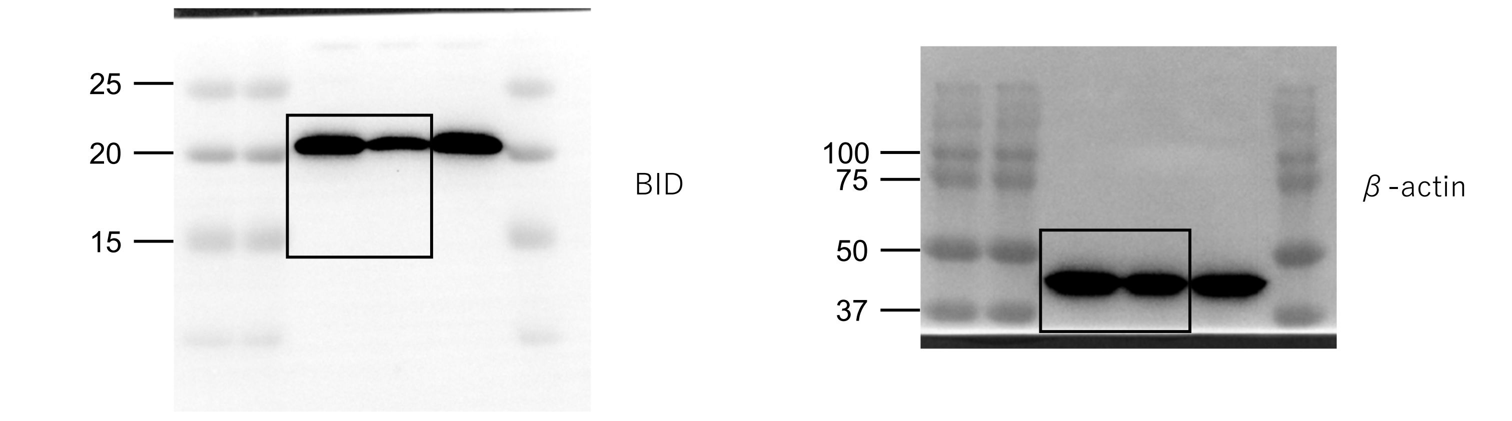

Supplemental Figure 11. Original immunoblot images merged with corresponding marker images.

Table S1. Oligo DNA sequences used for gene knockout, shRNA vector construction, and real-time PCR.

Designation	Sequence	Reference
**DNA sequence for pCas9 vectors**		
gTP53	5'-CACCGTCCATTGCTTGGGACGGCAA-3'	(1)
sgTFEB	5'- CACCGCATTGACAACATTATGCGTC-3'	CRISPR direct (2)
sgTFE3	5'- CACCGGCTGCAGGTGGTAGCGCGTT-3'	CRISPR direct (2)
sgATG5	5'-CACCGAAGAGTAAGTTATTTGACGT-3'	(3)
**DNA sequence for shRNA vectors**		
shNT	5'- ccggCAACAAGATGAAGAGCACCAActcgagTTGGTGCTCTTCATCTTGTTGtttttg -3'	
shBAK#1	5'- ccggTGGTACGAAGATTCTTCAAATctcgagATTTGAAGAATCTTCGTACCAtttttg -3'	
shBAK#2	5'- ccggCCCATTCACTACAGGTGAActcgagTTCACCTGTAGTGAATGGGtttttg -3'	
shBAX#3	5'- ccggCGAGTGGCAGCTGACATGTTTctcgagAAACATGTCAGCTGCCACTCGtttttg -3'	
shBAX#4	5'- ccggGCCGGAACTGATCAGAACCATctcgagATGGTTCTGATCAGTTCCGGCtttttg -3'	
shBID#1	5'- ccggCTTTCACACAACAGTGAATTTctcgagAAATTCACTGTTGTGTGAAAGtttttg -3'	
shBID#2	5'- ccggGTGAGGAGCTTAGCCAGAAATctcgagATTTCTGGCTAAGCTCCTCACtttttg -3'	
**DNA sequence for real-time PCR**		
hCASP8 Fwd	5'- AGAAGAGGGTCATCCTGGGAGA-3'	Harvard Primer Bank
hCASP8 Rev	5'- TCAGGACTTCCTTCAAGGCTGC-3'	
hBAX Fwd	5'- CCCGAGAGGTCTTTTTCCGAG-3'	Harvard Primer Bank
hBAX Rev	5'- CCAGCCCATGATGGTTCTGAT-3'	
hBAK Fwd	5'- CATCAACCGACGCTATGACTC-3'	Harvard Primer Bank
hBAK Rev	5'- GTCAGGCCATGCTGGTAGAC-3'	
hBID Fwd	5'- ACTGGTGTTTGGCTTCCTCC-3'	(4)
hBID Rev	5'- ATTCTTCCCAAGCGGGAGTG-3'	

References

1 Li, L., Mao, Y., Zhao, L., Li, L., Wu, J., Zhao, M. *et al.* p53 regulation of ammonia metabolism through urea cycle controls polyamine biosynthesis. Nature. 2019;567:253-256.

2 Naito, Y., Hino, K., Bono, H. & Ui-Tei, K. CRISPRdirect: software for designing CRISPR/Cas guide RNA with reduced off-target sites. Bioinformatics. 2015;31:1120-1123.

3 O'Prey, J., Sakamaki, J., Baudot, A. D., New, M., Van Acker, T., Tooze, S. A. *et al.* Application of CRISPR/Cas9 to Autophagy Research. Methods Enzymol. 2017;588:79-108.

4 Li, X., Zhao, Y., Xia, Q., Zheng, L., Liu, L., Zhao, B. *et al.* Nuclear translocation of annexin 1 following oxygen-glucose deprivation-reperfusion induces apoptosis by regulating Bid expression via p53 binding. Cell death & disease. 2016;7:e2356.
